# Future geographical distribution of *Aedes albopictus* in China under climate change scenarios

**DOI:** 10.1371/journal.pone.0327818

**Published:** 2025-08-06

**Authors:** Jianjun Xu, Rulin Wang, Zhenhan Mo, Han Zhang, Yujing Zhang

**Affiliations:** 1 Department of Hospital - Acquired Infection Management, Chengdu Integrated TCM & Western Medicine Hospital, Chengdu, Sichuan, PR China; 2 Water-Saving Agriculture in Southern Hill Area Key Laboratory of Sichuan Province, Chengdu, Sichuan, PR China; 3 Sichuan Provincial Rural Economic Information Center, Chengdu, Sichuan, PR China; National Institute of Malaria Research, INDIA

## Abstract

Amidst the escalating global threat of dengue fever, the distribution of its primary vector, *Aedes albopictus*, is undergoing significant shifts due to climate change. This study utilized Biomod2 to simulate the distribution changes of *Ae. albopictus* in China under future climate scenarios, providing critical insights for public health preparedness. Results showed that, the ensemble model achieved an ROC value of 0.968, a TSS value of 0.81, and a KAPPA value of 0.789, indicating high accuracy. Under current climate condition, the highly suitability regions were predominantly in the southern and eastern coastal areas of China. Guangdong, Guangxi, and Hunan possessed the largest areas of highly suitability, measuring 15.61 × 10^4^ km^2^, 20.84 × 10^4^ km^2^ and 11.71 × 10^4^ km^2^, respectively. Under SSP1–2.6 in the 2050s, highly suitability regions were projected to expand significantly, particularly in central Guangxi, northern Guangdong, and central Fujian. Centroids of the total suitability regions were predicted to shift southeast under SSP1–2.6 and SSP5–8.5, and northeast under SSP2–4.5 and SSP3–7.0, reflecting the dynamic response of *Ae. albopictus* to climate change. These findings underscore the imperative for climate-adaptive strategies in public health policies to mitigate the risks of dengue fever transmission in China.

## 1 Introduction

*Aedes albopictus* (Diptera: Culicidae) is widely distributed across China, spanning tropical to temperate zones from Hainan to Shenyang and Dalian in the north, Longxian and Baoji in the west, and Tibet in the southwest [1,2]. Recent studies highlight its seasonal adaptability: winter distributions are confined to southern subtropical areas, while summer ranges extend to northeastern China and the eastern Qinghai-Tibet Plateau, driven by transient warm and humid conditions [3]. This extensive range establishes *Ae. albopictus* as the primary vector for dengue fever in most regions of the country, with its ecological adaptability enabling colonization of both urban and rural habitats. Spatiotemporal analyses of dengue outbreaks from 2005 to 2017 revealed significant clustering in Guangdong and Yunnan provinces, where environmental factors such as annual minimum temperature and precipitation synergistically drive transmission risks [1,2]. Over the past three decades, China has witnessed a marked expansion of dengue-affected areas, from southern coastal regions to central provinces like Henan, driven by climate warming, urbanization, and increased human mobility [4]. Notably, imported cases from Southeast Asia—accounting for 70.8% of international introductions—have repeatedly triggered local epidemics, highlighting the vulnerability of regions with established mosquito populations [4]. Vector-borne diseases, such as dengue fever, Zika virus, and yellow fever, pose significant threats to global public health [5]. The spread of these diseases is influenced by a variety of factors [6], among which meteorological elements play a crucial role [7]. Meteorological elements include temperature, humidity, precipitation, wind speed, and air pressure, which directly or indirectly affect the ecological habits of mosquitoes, life cycles, and the survival and transmission capabilities of pathogens [5,7]. Climate change significantly impacts mosquito vector distribution and activity. Studies show that temperature and precipitation are key determinants of mosquito population dynamics and disease transmission. For example, Lu et al. developed a Species-specific Suitable Conditions Index (SCI) to assess dengue transmission risk in Guangdong, linking higher SCI values for Aedes aegypti to increased transmission [8]. Similarly, Feng et al. emphasized the role of interdisciplinary collaboration under the One Health framework in predicting dengue outbreaks [9]. The One Health approach, which recognizes the interconnectedness of human, animal, and environmental health systems, provides a critical framework for addressing vector-borne disease challenges under climate change. This paradigm emphasizes integrated surveillance of climatic variables, vector ecology, and human population dynamics – elements particularly relevant to *Ae. albopictus*’ range expansion. By bridging epidemiological models with ecological projections, One Health strategies can optimize early warning systems and coordinate interventions across public health, urban planning, and environmental management sectors [10]. Globally, Laporta et al. projected shifts in *Aedes* species distribution under climate change, indicating potential expansion into temperate regions [11]. These insights highlight the need for adaptive strategies to address dengue risks in China.

Temperature is one of the key factors influencing the spread of vector-borne diseases. The development, reproduction, and activity of mosquitoes are closely related to ambient temperature [12,13]. Within an optimal temperature range, the breeding speed of mosquitoes accelerates, and the efficiency of viral replication increases, thereby raising the potential risk of disease transmission [14,15]. However, temperatures that are too high or too low can inhibit the growth of mosquitoes and the activity of the virus [16]. For *Ae. albopictus*, temperature is particularly critical for larval development, with warmer conditions typically shortening the aquatic life stage and promoting population expansion. Humidity also has a significant impact on vector-borne diseases. A high-humidity environment is conducive to the development of mosquito adults, which live longer in such conditions, increasing the chances of blood feeding and disease transmission [16,17]. Adult *Ae. albopictus* prefer dark and sheltered resting locations, with peak blood-feeding activity occurring during early morning and late afternoon. Precipitation provides the water sources needed for mosquito breeding, and areas with standing water become hotspots for mosquito reproduction and high-risk areas for disease transmission [18–20]. However, it should be noted that in many small breeding sites, the water does not come from rain but from human activity, especially in places with low rainfall. Therefore, the rainfall variable may not work well in models developed for dry areas/ecosystems. As a container-breeding species, *Ae. albopictus* thrives in artificial and natural water-holding habitats, making rainfall patterns a key determinant of its distribution [3,12]. Wind speed and air pressure changes directly affect the flying ability and range of mosquitoes. Changes in wind speed can affect their frequency of contact with hosts [21]. Additionally, the transmission capacity of *Ae. albopictus* is influenced by both its biological traits and environmental factors. Susceptibility to dengue virus varies among geographic strains, potentially due to differences in virus titer, serotype, virulence, and local climatic conditions. Thus, the interaction between mosquito ecology and environmental variables ultimately shapes disease transmission risks.

Based on the R software, the Biomod2 is well-known and widely used due to its inclusion of 10 commonly used species distribution models and its free and open accessibility, making it the most mature multi-model platform to date [22–25]. This study applies Biomod2 to simulate the geographical distribution of *Ae. albopictus* in China under future climate change scenarios, with the aim of providing a basis for relevant departments to formulate effective monitoring measures and reasonable control methods.

## 2 Materials and methods

### 2.1 Distribution data of *Aedes albopictus*

**Fig 1:**
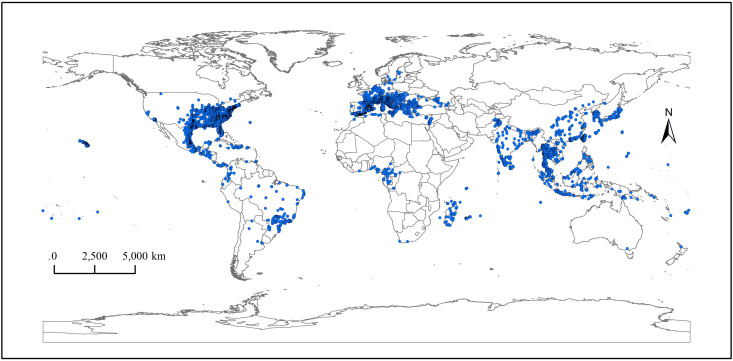
Distribution data of *Ae. albopictus* in the world. The boundary was obtained from Natural Earth (http://www.naturalearthdata.com/). Based on the principle of national and territorial integrity, we have modified and adjusted the vector boundary.

The distribution data of *Ae. albopictus* mainly come from the Global Biodiversity Information Facility database, the China National Knowledge Infrastructure, Web of Science, PubMed, Medline, and other online database publications of relevant literature. ENMTools was used to proofread and screen the obtained distribution points, excluding the impact of overfitting simulation caused by high spatial correlation [26–29], and a total of 10963 points were obtained (Fig 1).

### 2.2 Environmental variables

By reviewing relevant literature, 19 bioclimatic factors related to temperature and precipitation were selected data from the global climate dataset WorldClim (https://www.worldclim.org) (Table 1). These include current climate data (1970–2000) and future climate data (2050s, 2070s), with a spatial resolution of 2.5 arc-minutes [30]. The future climate data is based on the medium-resolution climate system model (BC-CSM2-MR) from the China (Beijing) Climate Center for the sixth Coupled Model Intercomparison Project (CMIP6), which is most suitable for China. The climate projections are based on four Shared Socioeconomic Pathways (SSP). SSP1–2.6 envisions a sustainable world with green energy, low population growth, and reduced fossil fuel use. SSP2–4.5 assumes moderate population and economic growth with current energy trends. SSP3–7.0 depicts a fragmented world with high population growth, slow economic progress, and limited cooperation. SSP5–8.5 projects high population growth, heavy fossil fuel reliance, and severe climate impacts [31,32].

**Table 1:** List of 19 bioclimatic factors.

Variable	Description
bio1	Annual Mean Temperature
bio2	Mean Diurnal Range (Mean of monthly (max temp – min temp))
bio3	Isothermality (BIO2/BIO7) (×100)
bio4	Temperature Seasonality (standard deviation ×100)
bio5	Max Temperature of Warmest Month
bio6	Min Temperature of Coldest Month
bio7	Temperature Annual Range (BIO5-BIO6)
bio8	Mean Temperature of Wettest Quarter
bio9	Mean Temperature of Driest Quarter
bio10	Mean Temperature of Warmest Quarter
bio11	Mean Temperature of Coldest Quarter
bio12	Annual Precipitation
bio13	Precipitation of Wettest Month
bio14	Precipitation of Driest Month
bio15	Precipitation Seasonality (Coefficient of Variation)
bio16	Precipitation of Wettest Quarter
bio17	Precipitation of Driest Quarter
bio18	Precipitation of Warmest Quarter
bio19	Precipitation of Coldest Quarter

### 2.3 Model construction

The distribution modeling of *Ae. albopictus* was conducted using the Biomod2 software package [22], which involved Random Forest (RF), Artificial Neural Networks (ANN), Generalized Linear Models (GLM), Boosted Regression Tree Models (BRT), Classification and Regression Tree Models (CART), Surface Range Envelope Models (SRE), Flexible Discriminant Analysis (FDA) and Maximum Entropy Models (MaxEnt). The MaxEnt model was calibrated using optimized parameters from the ENMeval package, with a regularization multiplier (RM) set to 0.5 and the feature combination designated as LQ, while the default model parameters of Biomod2 were adopted for the remaining models [33]. 75% of the distribution points were randomly selected as the training dataset. To avoid errors associated with a single modeling event, each model underwent the aforementioned process 10 times, resulting in a total of 80 modeling outcomes [23,25].

The importance of each environmental factor was evaluated through Biomod2, and the accuracy of each individual model was assessed using the Receiver Operating Characteristic (ROC) curve and the True Skill Statistic (TSS). The Area Under the Curve (AUC) values ranged from 0.5 to 1.0, where 0.5 represents completely random classification, and 1.0 indicates perfect classification. An AUC value greater than 0.8 suggests that the model has good or very good performance, while a value below 0.7 indicates poor predictive results. The TSS value, which ranges from 0 to 1, represents the net prediction success rate on measured samples. When the TSS value was greater than 0.7, it indicated a high prediction accuracy, while the value was less than 0.5 suggests a low accuracy. KAPPA is an index for measuring the consistency of classification models. It assesses the consistency between model predictions and actual observations. Its values typically range from −1–1, with higher values indicating better consistency. A KAPPA value greater than 0.7 is generally considered to reflect “good” consistency [34]. Based on the evaluation results of the individual models, the five modeling outcomes with the highest TSS, KAPPA and AUC values were selected to construct an ensemble model [22,24].

## 3 Results

### 3.1 Model accuracy

**Fig 2:**
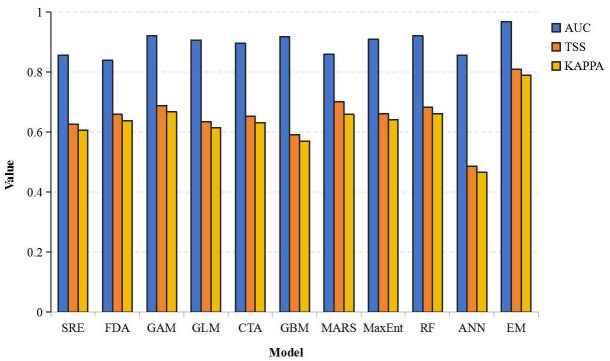
Evaluation index of individual model and ensemble model in Biomod2.

When compared to individual models (Fig 2), the ensemble model demonstrated superior performance, achieving an ROC value of 0.968, a TSS value of 0.81, and a KAPPA value of 0.789, indicating that the ensemble model provides a more accurate and credible prediction of the potential suitable distribution of *Ae. albopictus*.

### 3.2 Suitability region of *Ae. albopictus* under current climate situation

**Fig 3:**
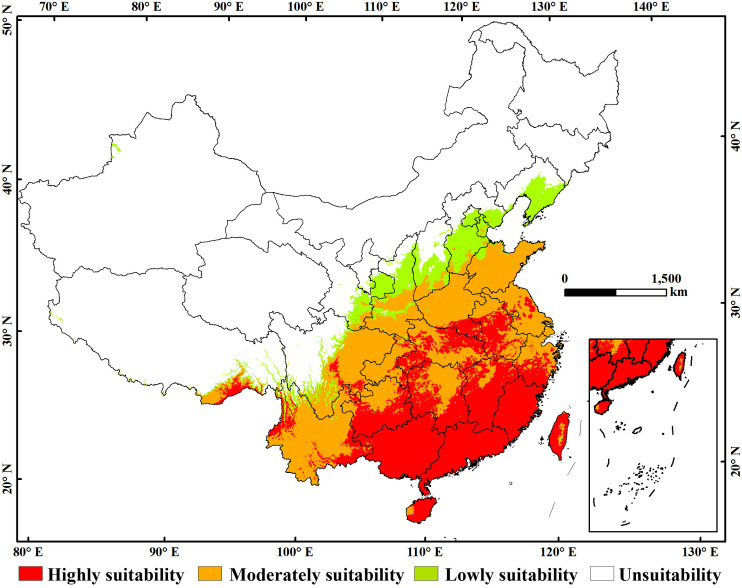
Suitability region of *Aedes albopictus* in China under current climate situation. The boundary was obtained from Natural Earth (http://www.naturalearthdata.com/). Based on the principle of national and territorial integrity, we have modified and adjusted the vector boundary.

The highly suitability regions of *Ae. albopictus* in China were predominantly concentrated in the warm and humid southern and eastern coastal areas, providing an optimal environment for its survival. Specifically, Guangdong, Guangxi, and Hunan possessed the largest areas of highly suitability, measuring 15.61 × 10^4^ km^2^, 20.84 × 10^4^ km^2^ and 11.71 × 10^4^ km^2^, respectively. Additionally, Jiangsu, Anhui, Sichuan, Hubei, Chongqing, Zhejiang, Hunan, Jiangxi, Yunnan, Guizhou, Fujian, Guangxi, Taiwan, and Hainan also had varying degrees of highly suitability regions, ranging from 1.18 × 10^4^ km^2^ to 6.79 × 10^4^ km^2^. Collectively, the area of highly suitability regions in China was 121.77 × 10^4^ km^2^, which constituted a substantial proportion of the total suitability regions (Fig 3).

Quantitatively, the area of moderately suitability regions was 143.40 × 10^4^ km^2^. Notably, Hebei, Shanxi, and Shaanxi provinces exhibited the largest measuring 1.52 × 10^4^ km^2^, 2.47 × 10^4^ km^2^ and 9.47 × 10^4^ km^2^, respectively. Tianjin, Jiangsu, Anhui, Sichuan, Hubei, Chongqing, Shanghai, Zhejiang, Hunan, Jiangxi, Yunnan, Guizhou, Fujian, Guangxi, Taiwan, and Hainan also contained moderately suitability regions, with areas ranging from 0.11 × 10^4^ km^2^ to 24.94 × 10^4^ km^2^. although these regions are not the most ideal for *Ae. albopictus,* they still pose a risk for the establishment and proliferation of its populations (Fig 3).

### 3.3 Changes in spatial distribution patterns of the suitability regions under climate change scenarios

**Fig 4:**
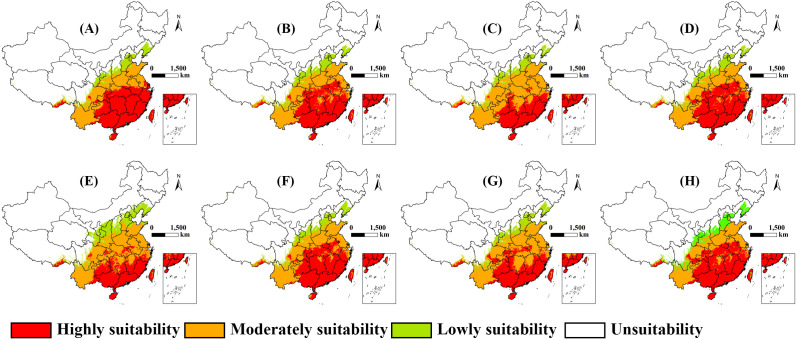
Potential suitability region of *Aedes albopictus* in China under climate change scenarios. **(A) SSP1-2.6 in 2050s; (B) SSP2-4.5 in 2050s; (C) SSP3-7.0 in 2050s; (D) SSP5-8.5 in 2050s; (E) SSP1-2.6 in 2090s; (F) SSP2-4.5 in 2090s; (G) SSP3-7.0 in 2090s; (H) SSP5-8.5 in 2090s.** The boundary was obtained from Natural Earth (http://www.naturalearthdata.com/). Based on the principle of national and territorial integrity, we have modified and adjusted the vector boundary.

Fig 4 illustrated that the distribution of suitability regions varies greatly under different scenarios. For instance, under SSP1–2.6 scenario in 2050s, both highly and moderately suitability regions showed a significant expansion. The expanded highly suitability regions were located in central Guangxi, northern Guangdong and central Fujian, while the expanded moderately suitability regions were observed in Anhui, Hubei, Hunan, Guizhou, Zhejiang, Eastern Sichuan and Western Chongqing. On the contrary, under SSP3–7.0 scenario, a substantial portion of the highly suitability regions in Guangxi, Guangdong and Fujian were projected to transition to moderately.

**Fig 5:**
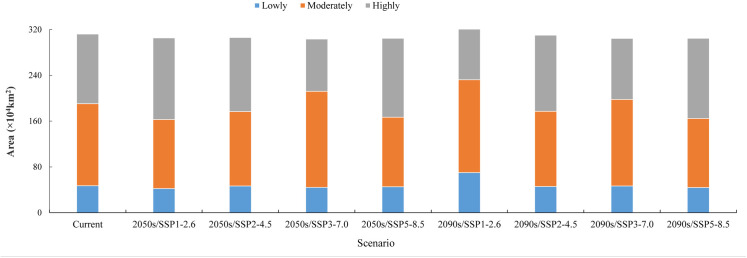
Statistics of changes in suitability regions.

Under SSP1–2.6 scenario, the area of highly suitability regions was projected to initially increase from 121.77 × 10^4^ km^2^ (current) to 142.76 × 10^4^ km^2^ (2050s) (+17.2%) and then decrease to 101.33 × 10^4^ km^2^ (2090s) (−16.8%). The area of moderately suitability regions was expected to first decrease from 143.39 × 10^4^ km^2^ (current) to 120.65 × 10^4^ km^2^ (2050s) (−15.9%) and then increase to 162.19 × 10^4^ km^2^ (2090s) (+13.1%). The area of lowly suitability regions would initially decrease from 47.12 × 10^4^ km^2^ (current) to 42.09 × 10^4^ km^2^ (2050s) (−10.7%) and then increase to 70.03 × 10^4^ km^2^ (2090s) (+48.6%) (Figs 4 and 5).

Under SSP2–4.5 scenario, the area of highly suitability regions was anticipated to increase from 121.77 × 10^4^ km^2^ (current) to 129.41 × 10^4^ km^2^ (2050s) (+6.3%) and 133.02 × 10^4^ km^2^ (2090s) (+9.2%). The area of moderately suitability regions would decrease from 143.39 × 10^4^ km^2^ (current) to 130.11 × 10^4^ km^2^ (2050s) (−9.3%) and 131.22 × 10^4^ km^2^ (2090s) (−8.5%). The area of the lowly suitability region was expected to decrease from 47.12 × 10^4^ km^2^ (current) to 46.68 × 10^4^ km^2^ (2050s) (−0.9%) and 45.86 × 10^4^ km^2^ (2090s) (−2.7%) (Figs 4 and 5).

Under SSP3–7.0 scenario, the area of highly suitability regions was predicted to decrease from 121.77 × 10^4^ km^2^ (current) to 91.36 × 10^4^ km^2^ (2050s) (−25%) and 106.49 × 10^4^ km^2^ (2090s) (−12.6%). The area of moderately suitability regions would increase from 143.39 × 10^4^ km^2^ (current) to 167.88 × 10^4^ km^2^ (2050s) (+17.1%) and 151.36 × 10^4^ km^2^ (2090s) (+5.6%). The area of lowly suitability regions was expected to decrease from 47.12 × 10^4^ km^2^ (current) to 44.1 × 10^4^ km^2^ (2050s) (−6.4%) and 46.62 × 10^4^ km^2^ (2090s) (−1.1%) (Figs 4 and 5).

Under SSP5–8.5, the area of highly suitability region was expected to increase from 121.77 × 10^4^ km^2^ (current) to 138.05 × 10^4^ km^2^ (2050s) (+13.4%) and 140.05 × 10^4^ km^2^ (2090s) (+15%). The area of the moderately suitability region would decrease from 143.39 × 10^4^ km^2^ (current) to 121.57 × 10^4^ km^2^ (2050s) (−15.2%) and 120.66 × 10^4^ km^2^ (2090s) (−15.9%). The area of the lowly suitability region was projected to decrease from 47.12 × 10^4^ km^2^ (current) to 45.24 × 10^4^ km^2^ (2050s) (−4%) and 43.95 × 10^4^ km^2^ (2090s) (−6.7%) (Figs 4 and 5).

### 3.4 Centroid migrations of suitability regions under climate change scenarios

**Fig 6:**
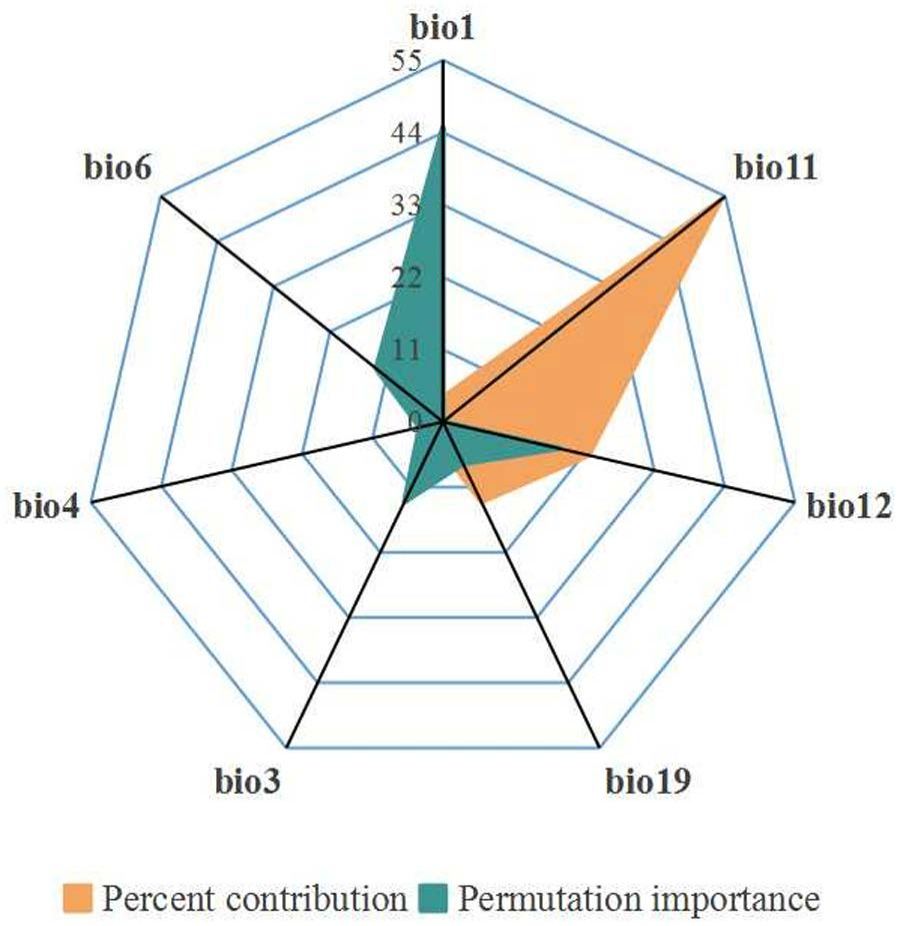
Centroid migrations of suitability regions under climate change scenarios.

Under SSP1–2.6 scenario, the centroid of the total suitability region was projected to shift southeast from 110.94°E/28.61°N (current) to 111.01°E/28.59°N (2050s) by 6.63 km, and then northwest by 5.99 km to 110.95°E/28.6°N (2090s) (Fig 6). Overall, from current to 2090s, the centroid was expected to migrate 1.44 km southeast. Under SSP2–4.5, the centroid was anticipated to migrate southeast from 110.94°E/28.61°N (current) to 111.01°E/28.53°N (2050s) by 10.74 km, and then northeast by 15.13 km to 111.06°E/28.67°N (2090s) (Fig 6). From current to 2090s, the centroid was expected to migrate 13.46 km northeast. Under SSP3–7.0, the centroid was expected to migrate southeast from 110.94°E/28.61°N (current) to 111.01°E/28.51°N (2050s) by 12.29 km, and then further southeast by 5.04 km to 111.07°E/28.52°N (2090s) (Fig 6). From current to 2090s, the centroid was projected to migrate 15.78 km southeast. Under SSP5–8.5, the centroid was predicted to migrate southeast from 110.94°E/28.61°N (current) to 111.05°E/28.54°N (2050s) by 12.87 km, and then northeast by 5.87 km to 111.07°E/28.59°N (2090s) (Fig 6). Overall, from current to 2090s, the centroid was expected to migrate 13.31 km southeast.

### 3.5 Environmental variables

**Fig 7:**
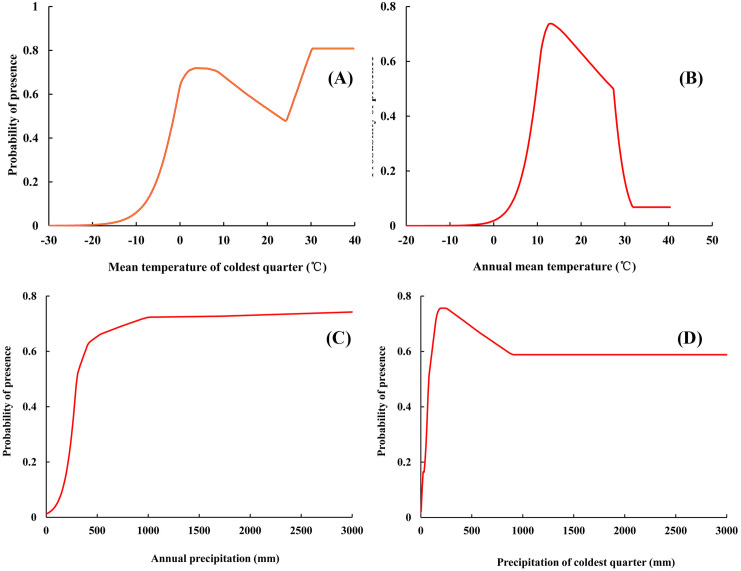
Percent contribution and permutation importance of environmental variables.

For *Ae. albopictus,* the top three variables with the higher percent contribution rate were the mean temperature of coldest quarter (bio11, 54.12%), the annual precipitation (bio12, 22.76%) and the precipitation of coldest quarter (bio19, 13.47%). From the perspective of permutation importance, the top four variables were the annual mean temperature (bio1, 44.77%), the annual precipitation (bio12, 18.09%), the isothermality (bio3, 13.46) and the min temperature of coldest month (bio6, 13.01%) (Fig 7).

**Fig 8:**
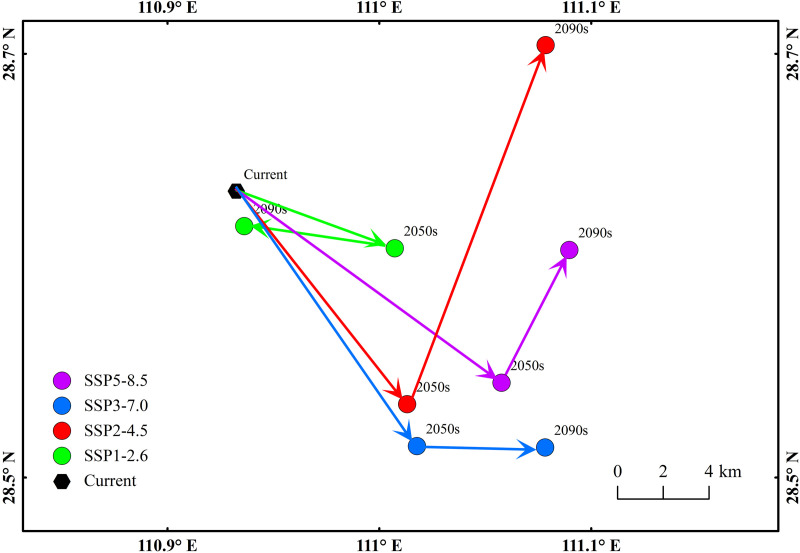
Response curves between environmental variables and presence probability.

According to the response curve between environmental variables and presence probability, the suitable range of environmental variables for the distribution of *Ae. albopictus* can be determined. When the value of the mean temperature of coldest quarter was higher than −3.21°C, it was suitable for the distribution of *Ae. albopictus*. When the value of annual mean temperature was in the range of 8.34°C-13.06°C, with the increase in temperature, the predicted distribution probability increased, and decreased rapidly when the temperature was higher than 27.41°C. When the value of the annual precipitation was lower than 266.57 mm, the presence probability of *Ae. albopictus* was lower than 0.33, and the probability gradually increased with the precipitation (Fig 8).

## 4 Discussion

The global climate is changing rapidly, and the suitable areas for *Ae. albopictus* are also changing. Timely update is of great significance for the prevention and control of *Ae. albopictus* with the change of climate. This study collected the latest comprehensive monitoring data of *Ae. albopictus* in the world, predicted its geographical distribution range in China by Biomod2, and estimated the impact of climate change on its distribution under the SSPs scenarios. By comparing the prediction results of each individual model with the actual distribution of *Aedes albopictus*, as well as the AUC and TSS values, it was found that the RF, GBM, MaxEnt, GLM, CTA all achieved better results. The prediction accuracy of the ensemble model was significantly improved compared with the 10 individual models. The AUC value and TSS value increased by 7.4% and 19.8% respectively compared with the RF,. At the same time, the ensemble model also solved the problem that some species distribution models did not have high accuracy in describing the details of species’ suitability regions [35]. The above showed that the simulation of *Aedes albopictus* using the ensemble model is more accurate than individual models.

Climate is the most important factor determining species distribution on the planet. Climate change influences the stability of ecological frameworks and the diversity of the organism population, and changes in the species distributions are the clearest and most direct indication of climate change [36–38]. Global warming may fundamentally alter the framework and functions of land ecological systems, leading to changes in the inhabitable areas of multiple species and probably the acceleration of diversity loss [39,40]. Recent studies, such as Ren and Xu [41], emphasize that imported dengue cases are the primary driver of local epidemics in China, with climatic suitability (e.g., temperature and humidity) modulating the spillover risk. This aligns with our findings that the centroid shifts of *Ae. albopictus* suitability regions under SSP scenarios reflect dynamic responses to temperature and precipitation changes. Historically, *Ae. albopictus* was thought to be confined to southern China below 22°N [42,43]. However, recent studies indicate that the distribution of *Ae. albopictus* has expanded significantly across China, extending to northern and western regions such as Beijing, Shanxi, Shaanxi, and Gansu due to factors such as climate change, urbanization, and human mobility [1]. Li et al. highlighted that *Ae. albopictus* exhibits strong adaptability to urbanization, with construction land contributing 46.7% to its distribution model, suggesting synergistic effects between climate warming and anthropogenic landscapes in driving range shifts [44]. The species has been detected in nearly one-third of Chinese provinces, with its range extending to temperate regions [4,45–47]. Referring to the method proposed by Yue et al. [48], we calculated the centroids of inhabitable regions to identify the migration at different periods and scenarios, which reflects the response of *Aedes albopictus* to climate change. The results showed that the centroids would move to the northeast by the 2090s, and the area of the total inhabitable regions would expand. This finding is consistent with Liu et al. [49], who reported a significant northward expansion of *Ae. albopictus* in China, extending to provinces such as Beijing, Shanxi, and Shaanxi. Similarly, Wu et al. documented the species’ presence in previously unrecorded areas of Gansu and Shaanxi provinces, indicating a clear trend of range expansion driven by climate change [50]. However, discrepancies exist in the predicted extent of expansion. While our study projects a substantial increase in suitable areas under SSP1–2.6 and SSP5–8.5 scenarios, Liu et al. (2023) suggest a more gradual expansion, possibly due to differences in model inputs and climate projections [49].

Temperature is a critical environmental factor that affects mosquitoes’ development and reproduction. *Ae. albopictus*, being ectothermic, relies on ambient temperature for metabolic processes. Studies show that 10–35°C is optimal for most mosquitoes, with larval development peaking at ~28°C and ceasing below 10°C [12,51–53]. Ding et al. (2018) identified temperature suitability as the most influential factor in global *Aedes* distribution models, consistent with our variable importance analysis where the mean temperature of the coldest quarter (bio11) contributed 54.12% [41]. Liu et al. emphasized that minimum winter temperatures above −3.21°C significantly enhance overwintering survival, which aligns with our variable importance analysis identifying cold quarter mean temperature (bio11) as the top contributor [54]. In this study, the importance of the variables was determined using the Jackknife test method, and the most important environmental variables affecting *Ae. albopictus* mainly included the mean temperature of coldest quarter (bio11), the annual precipitation (bio12) and the precipitation of coldest quarter (bio19). Li et al. [51] found that *Ae. albopictus* cannot complete generational development below 15 °C, but can develop normally at 20–35 °C, and the development cycle shortens with increasing temperature.

Combining Fig 2, *Ae. albopictus* was concentrated in the southern, southwestern, southeastern, and central regions of China. These regions were mostly characterized by subtropical monsoon climate (with an mean temperature of 0–15 °C in the coldest month and above 22 °C in the warmest month) and temperate monsoon climate (with an mean temperature below 0 °C in the coldest month and above 22 °C in the warmest month), which were suitable for mosquito reproduction and development [55]. Response curves showed that the suitable range of bio11 and bio1 was >−3.21°C and 8.34°C-13.06°C, respectively, which were consistent with the above climate characteristics. According to a study by Servadio et al., there is a significant parabolic association between maximum average monthly temperature and the risk of mosquito-borne disease outbreaks in South and Southeast Asia, with outbreak risk peaking near 33.5°C [56]. This aligns with our findings, which highlight the importance of temperature in shaping the distribution and activity of *Ae. albopictus*. Similarly, Servadio et al. support the notion that climate change can lead to shifts in the geographic regions affected by vector-borne diseases rather than simple range expansions [56]. This underscores the need for adaptive public health strategies to address the changing patterns of disease transmission.

A drop in air pressure often indicates the approach of adverse weather conditions, which can influence mosquito behavior and human outdoor activities, thereby affecting the dynamics of disease spread. Under the backdrop of climate change, the increasing frequency of extreme weather events, such as droughts, floods, and hurricanes, brings new challenges to the transmission patterns of vector-borne diseases. These extreme events may alter the distribution and behavior of mosquitoes, increasing the uncertainty and complexity of disease outbreaks. Studies have shown that environmental humidity can affect the growth and development of insects [57]. The tolerance to humidity was relatively high, and *Ae. albopictus* can develop normally at a relative humidity of 85–95% [58], and the distribution of *Ae. albopictus* was positively correlated with humidity [59]. He et al. [60] found that the survival time of female adult *Ae. albopictus* increases with humidity, and the humidity effect is more significant under low temperature conditions. In addition, humidity was one of the important factors in the search for breeding sites for *Ae. albopictus*. An increase in precipitation can expand the breeding area of *Ae. albopictus* larvae and form new breeding grounds, thereby expanding their distribution range. The conclusion of this study pointed out that when the annual precipitation was higher than 266.57 mm, it was suitable for the distribution of *Ae. albopictus*, which indicated that higher humidity was beneficial for *Ae. albopictus.* This was consistent with the findings of some earlier studies. Overall, precipitation and temperature together limit the distribution pattern of *Ae. albopictus*.

## 5 Conclusions

This study employed an ensemble modeling approach to project the future geographical distribution of *Ae. albopictus* in China under climate change scenarios. Key findings revealed significant shifts in habitat suitability, driven primarily by temperature and precipitation changes. The ensemble model demonstrated high predictive accuracy (ROC = 0.968, TSS = 0.81), confirming its reliability in mapping the species’ ecological niche. Under current climatic conditions, highly suitable regions are concentrated in southern and eastern coastal provinces, with Guangdong, Guangxi, and Hunan identified as critical hotspots. By the 2050s and 2090s, climate-driven expansions or contractions of suitable areas were projected across all SSP scenarios, with notable southeastward centroid shifts under SSP1–2.6 and SSP5–8.5, and northeastward shifts under SSP2–4.5 and SSP3–7.0. These spatial dynamics reflect the species’ sensitivity to warmer winters (bio11), annual precipitation (bio12), and regional temperature gradients.

The findings underscore that climate change will likely exacerbate dengue transmission risks in both historically endemic and newly suitable regions. Central Guangxi, northern Guangdong, and central Fujian are projected to face heightened suitability, necessitating prioritized surveillance and vector control. Conversely, provinces experiencing reduced suitability (e.g., parts of Guangxi under SSP3–7.0) may require adaptive strategies to address residual populations in fragmented habitats. The study highlights the synergistic role of urbanization and human mobility in amplifying climate-driven range shifts, as observed in recent expansions to temperate zones.

To mitigate future risks, climate-resilient public health policies must integrate dynamic distribution models, early warning systems, and cross-sectoral collaboration under the One Health framework. Limitations include uncertainties in climate projections and localized anthropogenic factors (e.g., water storage practices), which warrant further investigation. Future research should refine models with finer-scale environmental data and validate predictions through longitudinal field monitoring. By aligning control strategies with these projections, China can enhance preparedness against the evolving threat of *Ae. albopictus* and dengue fever.
